# Association of cardio-renal biomarkers and mortality in the U.S.: a prospective cohort study

**DOI:** 10.1186/s12933-023-01986-2

**Published:** 2023-09-29

**Authors:** Fan Yang, Mingsi Wang, Yuzhu Chen, Jianjun Wu, Yilan Li

**Affiliations:** 1 Department of Cardiology, the Second Affiliated Hospital of Harbin Medical University, Harbin, 150086 China; 2 Key Laboratory of Myocardial Ischemia, Ministry of Education, Harbin, 150086 China; 3 College of Health Management of Harbin Medical University, Harbin, 150076 China; 4 The Second Affiliated Hospital of Harbin Medical University, Harbin, 150086 China

**Keywords:** Diabetes, NHANES, Predict risk model, Cardiovascular mortality, Death burden

## Abstract

**Objective:**

Diabetes poses a significant threat to human health. There is a lack of large-scale cohort studies to explore the association between mortality risk and indicators beyond blood glucose monitoring in diabetic populations.

**Methods:**

Multivariable Cox proportional hazards regression models were performed to investigate the association of 13 blood biomarkers with mortality risk in the National Health and Nutrition Examination Survey (NHANES) and biomarker levels were log-transformed and correlated with mortality.

**Results:**

During a median follow-up of 7.42 years, 1783 diabetic patients were enrolled. Compared to traditional risk factors, the addition of hs-cTnT, hs-cTnI, NT-proBNP, creatinine, cystatin C, and β-2 microglobulin biomarkers increased the predictive ability for all-cause mortality by 56.4%, 29.5%, 38.1%, 18.8%, 35.7%, and 41.3%, respectively. However, the inclusion of blood glucose monitoring had no impact on the prediction of all-cause mortality. Compared with the 1st quartiles of creatinine and Cystatin C, the risk of diabetes mortality were higher in the highest quartiles (HR: 5.16, 95% CI: 1.87–14.22; HR: 10.06, 95% CI: 4.20-24.13).

**Conclusions:**

In the diabetic population, elevated plasma levels of hs-cTnT, hs-cTnI, NT-proBNP, creatinine, cystatin C, and β-2 microglobulin serve as robust and straightforward predictors of long-term mortality compared to blood glucose levels and HbA1c values. Creatinine and cystatin C stand out as more precise markers for predicting diabetes mortality prior to blood glucose monitoring.

**Supplementary Information:**

The online version contains supplementary material available at 10.1186/s12933-023-01986-2.

## Key points

**Question**: Is blood glucose monitoring the most effective predictor of long-term survival in the diabetic population?

**Findings**: Among 1783 diabetic patients, the addition of hs-cTnT, hs-cTnI, NT-proBNP, creatinine, cystatin C, and β-2 microglobulin biomarkers increased the predictive ability for all-cause mortality by 56.4%, 29.5%, 38.1%, 18.8%, 35.7%, and 41.3%, respectively. However, blood glucose monitoring had no impact on the prediction of all-cause mortality.

**Meaning**: Among individuals with diabetes, the utilization of plasma cardiac and renal biomarkers as primary predictors for overall mortality is recommended.

**Supplementary Information:**

The online version contains supplementary material available at 10.1186/s12933-023-01986-2.

## Introduction

In recent years, diabetes has emerged as a major global public health issue. It is reported that in 2021, diabetes affected approximately 714 million adults, with a projected increase in prevalence in the future [1]. Among this population, cardiovascular disease (CVD) stands as the primary cause of mortality, accounting for over half of all deaths. Long-term diabetes can lead to diabetic cardiomyopathy [2], which in turn leads to the occurrence of cardiovascular events [3, 4]. However, early intervention and treatment are hampered by the high proportion of undiagnosed diabetes cases. To tackle this pressing public health concern, researchers are progressively concentrating on pinpointing the primary mortality risk factors in diabetes patients. The pivotal progress in the field revolves around the organization of these factors into a comprehensive risk prediction scoring system [5, 6]. However, at least in the context of clinical practice, where our ability to predict 90% of future cardiovascular events remains challenging. The estimation of the “number needed to treat” to prevent a single cardiovascular event still frequently exceeds 100 individuals. This relatively high number could be attributed to several factors, including limited treatment effectiveness and suboptimal risk stratification, especially among the diabetic population, where this issue is particularly significant [7].

Recent advancements have highlighted the potential of biomarkers as valuable tools for assessing biological processes, predicting disease risk, and monitoring treatment efficacy. Elevated levels of novel biomarkers, including NT-proBNP, high-sensitivity cardiac troponin T (hs-cTnT), and cystatin C, have emerged as indicators of all-cause mortality [8], including coronary heart disease, heart failure, stroke, and sudden death. Other biomarkers, including propeptide of type II collagen, creatinine clearance rate, estimated glomerular filtration rate (eGFR), and blood urea, have also been explored for their ability to predict cardiovascular events in diabetic patients [9, 10]. However, the findings from these studies have been inconsistent, contributing to uncertainty in the field. We are earnestly pursuing a deeper exploration of the substantial impact of biomarkers on individual health status, and the identification of these cardiac and renal biomarkers is of paramount importance to healthcare professionals. This identification process is poised to facilitate the high precision of healthcare, as it enables the accurate discernment of diabetic patients most in need of attention and intervention, thereby reducing the incidence of cardiovascular events. By delving into the intricate relationship between individual blood sugar control and cardiac-renal function, we hold the promise of offering optimized genetic and lifestyle recommendations for the next generation, thus mitigating the risk of cardiovascular diseases and diabetes. This intergenerational health enhancement will have a positive impact on overall societal health and healthcare costs.

In this context, our research represents a significant and innovative investigation aimed at comprehensively exploring the relationship between various cardiovascular-renal biomarkers and mortality in diabetes patients. By conducting a comparative analysis of the relative prognostic value of these biomarkers, our objective is to elucidate the pivotal role that cardiovascular-renal biomarkers can play in predicting mortality among diabetes patients, as opposed to traditional diabetes biomarkers. Through the identification of the most effective biomarkers for risk stratification, our study has the potential to drive the development of improved tools for mitigating the high mortality rates associated with diabetes.

## Methods

### Study population

**Fig. 1:**
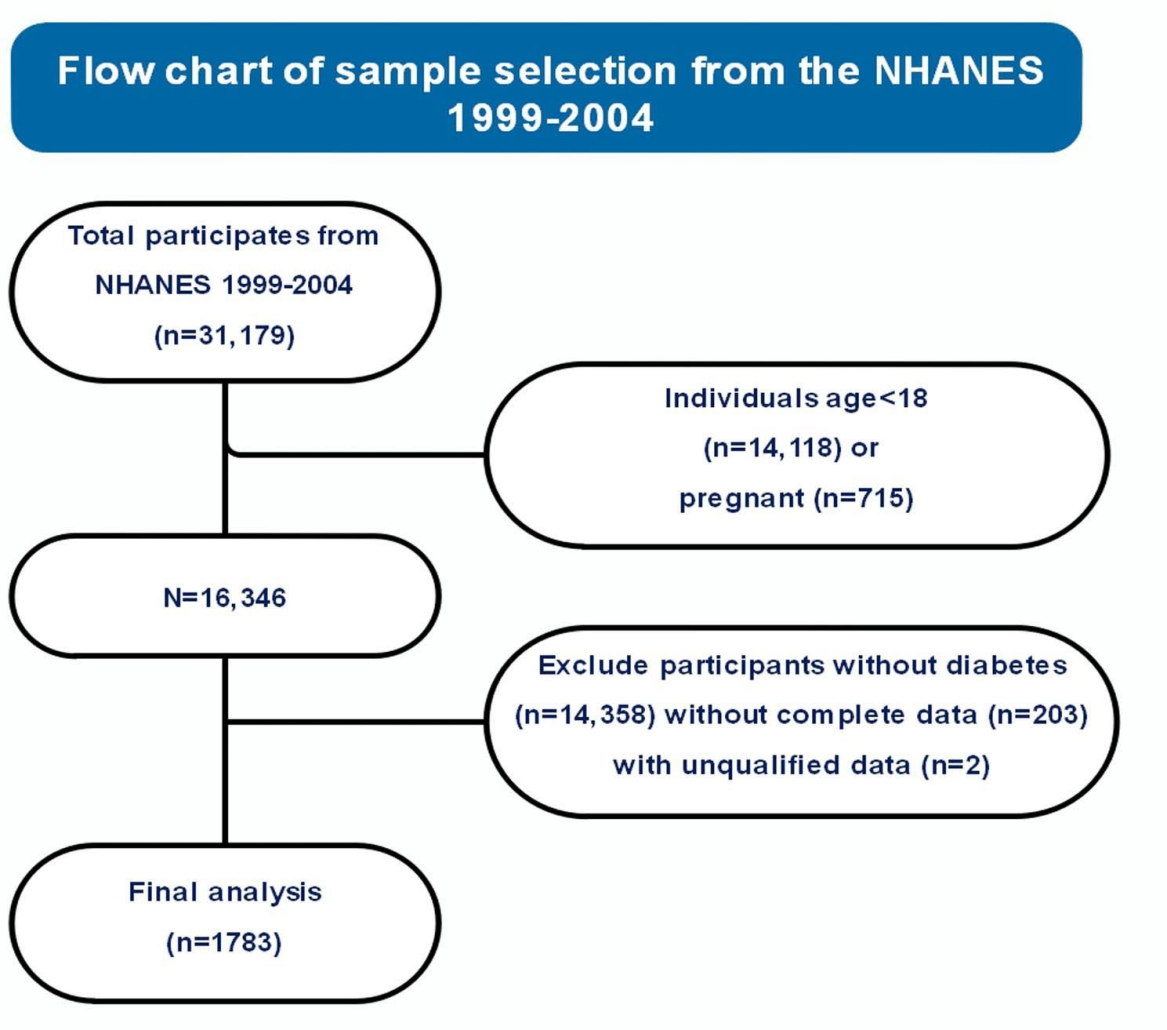
Flowchart of our study. Data were required from National Health and Nutrition Examination Survey (NHANES) data and NCHS mortality data with the linkage of 31,717 individuals obtained. Participants were included based on age, presence of diabetes, and availability of biomarker data, resulting in a final sample size of 1,783 individuals. Survey strata, and primary sample units were applied in our analysis procedure whenever feasible

The NHANES is a comprehensive cross-sectional survey administered by the National Center for Health Statistics. It is specifically designed to monitor the health and nutritional status of non-institutionalized civilians in the United States. Data collection for NHANES is an ongoing process, with information continuously gathered and made available to the public in two-year increments. For this manuscript, we utilized data from the 1999–2004 survey cycles. It is important to note that we combined data from these specific survey cycles to facilitate our research objectives. For a more comprehensive understanding of NHANES, including its sampling methodology, data collection procedures, and interview processes, detailed information is readily accessible on the NHANES website (http://www.cdc.gov/nchs/NHANES.htm). We enrolled a total of 31,179 individuals, and to ensure the control of diabetes severity, we established inclusion criteria. These criteria included participants who were over 18 years old and diabetes mellitus is defined based on the 2022 guidelines of the American Diabetes Association. It is characterized by a serum glycated hemoglobin level greater than 6.5%, serum fasting glucose level of ≥ 126 mg/dL, and/or self-reported use of anti-diabetic medications in conjunction with a confirmed physician diagnosis of diabetes. We also established exclusion criteria. Individuals who were under 18 years old (N = 14,118), pregnant women (N = 715), and those with missing data (N = 205) were excluded from the current analyses. Adolescents, being legally incapable of providing independent informed consent, may also introduce additional complexities in data interpretation and analysis due to their developmental stage and potential dependency on adults. The inclusion of pregnant women in the study could introduce additional complexity and confounding factors due to the physiological changes they undergo, including fluctuations in hormone levels and blood glucose levels, making the interpretation of research findings challenging. After applying these inclusion and exclusion criteria, our final analytical cohort consisted of 1,783 individuals with diabetes who had completed measurements and provided available follow-up data on mortality (Fig. 1).

### Ethics statement

This study utilizes publicly available data from the Third National Health and Nutrition Examination Survey, conducted by the National Center for Health Statistics (NCHS). NHANES provides publicly demographic and laboratory data and written informed consent for data collection was obtained from all subjects. The NHANES protocol was approved by the NCHS Research Ethics Review Board. Deidentified participant data and corresponding documentation can be accessed publicly online via the following link: https://www.cdc.gov/nchs/surveys.htm.

### Study variables

The following covariates were demonstrated in previous studies to be associated with mortality risk among diabetic patients for our analyses: gender (male and female), age (continuous), race (Mexican American, Non-Hispanic white, Non-Hispanic black and Other), body mass index (BMI, kg/m^2^), education level (less than high school, high school or equivalent, greater than high school), physical activity (never, moderate, vigorous), PIR index (≤ 1, 1.01–4.99, ≥ 5), drinking status (yes or no), self-reported history of CVD (yes or no), and chronic kidney disease (CKD) (yes or no). Physical activity is assessed by the number of moderate to high intensity activities (such as walking, jogging, running, swimming, cycling, dancing, or yard work) per week, while lack of physical activity is defined as never performing moderate or high-intensity activities. Categories of cotinine levels were (1) cotinine > 10 ng/mL, (2) limit of detection (LOD) -10 ng/mL, and (3) cotinine < LOD. Drinkers were defined as participants who drank at least 12 alcohol drinks in any given year. History of CVD in the NHANES was based on the self-reported data of a previous diagnosis of congestive heart failure, coronary heart disease, angina pectoris, heart attack, or stroke. The CKD Epidemiology Collaboration (CKD-EPI) equation was utilized to estimate the eGFR. CKD was defined as an estimated glomerular filtration rate (eGFR) < 60mL/min/1.73 m^2^. eGFR was calculated using the CKD-EPI equation: eGFR = 141 × min(Scr/κ, 1)α × max(Scr/κ, 1) − 1.209 × 0.993age × 1.018 [if female] × 1.159 [if Black], where Scr represents serum creatinine, κ is 0.7 for females and 0.9 for males, α is -0.329 for females and − 0.411 for males, min indicates the minimum of Scr/κ or 1, and max indicates the maximum of Scr/κ or 1 [11].

### Assessment of biochemistry indexes

Blood specimens were collected at the NHANES mobile examination centers (MECs). General information on specimen collection and quality control for laboratory data is available at http://www.cdc.gov/nchs/data/NHANES/NHANES_03_04/lab_c_generaldoc.pdf. Dichromatic digital endpoint method was used to measure serum and urinary albumin, while serum creatinine was assayed with a Hitachi 917 multichannel analyzer during NHANES 1999–2000 and NHANES 2001–2004 used the Jaffe rate method with the BeckmanSynchronLX20 modular chemistry analyzer.

### Ascertainment of death

The NDI provides a probabilistic score for each record that is matched by social security number, name, gender, race/nationality, date/state of birth, state of death, death certificate number, and date of death. The National Center for Health Statistics maintains the death certificates of individuals who have passed away, and both participant deaths and the causes of death are routinely updated until December 31st, 2019. We used the International Classification of Diseases, Tenth Revision (ICD-10) to define the underlying causes of death. CVD mortality was categorized by the NCHS as deaths caused by heart disease (ICD-10 codes I00-I09, I11, I13, and I20-I51) or cerebrovascular disease (ICD-10 codes I60-I69), while cancer mortality was defined as deaths caused by malignant neoplasms (ICD-10 codes C00-C97). This approach has been validated by the CDC and is commonly used in their reports. In our study, we considered all-cause mortality as any type of death attributed to a specific cause.

### Statistical analysis

In our analysis, conducted using R software version 4.0.4, we deemed statistical significance as a two-tailed p-value below 0.05. To address multiple testing, we applied false discovery rate (FDR) correction through the Fdr package. A corrected P value of less than 0.05 was considered statistically significant. In order to accommodate the complex survey procedure and design of the NHANES, we utilized the survey package to estimate the variance.

Baseline characteristics: descriptive statistics were used to summarize the continuous variables, presented as means with standard deviations (SDs) or medians with quartiles (Qs). Categorical variables were presented as frequencies and percentages. ANOVA or Kruskal-Wallis tests were used to determine differences among groups for continuous variables, and chi-square tests were used for categorical variables.

Poisson distribution: poisson regression models were utilized to estimate mortality rates per 1000 person-years and the corresponding 95% confidence intervals (CIs) for each major cause of death within each layer of serum biomarkers.

Kaplan-meier survival analysis: in this study, we focused on specific causes of death, including all-cause mortality, CVD mortality and diabetes mortality. We chose the Kaplan-Meier (KM) method over the Fine-Gray competing risks model as it was more suitable for this study. KM survival analysis was conducted to estimate the survival rate across different serum biomarker groups, with differences compared using the log-rank test.

Cox proportional hazards regression analysis: cox proportional hazards regression models were used to calculate hazard ratios (HRs) and 95% CIs to evaluate the association between serum biomarker levels and all-cause mortality, as well as CVD/all-cause/diabetes mortality, while controlling for potential confounders including gender, age, race, BMI, education, activity, PIR, cotinine, drinking, hypertension, hyperlipidemia, CVD, and CKD. We also employed cox proportional hazards models to compare two models based on akaike information criterion (AIC) and bayesian information criterion (BIC) to evaluate the proportional hazards assumption.

Correlation heatmap: we used pearson’s correlation analysis to investigate the associations between potential biomarkers related to aging and constipation. Correlation coefficients (r) ranged from − 1.0 to 1.0, with 0 indicating no correlation. We presented the results using a cross-correlation heatmap, where red indicated positive correlations, and blue indicated negative correlations.

Restricted cubic spline (RCS) analysis: RCS analysis was performed to examine the dose-response relationship between biomarkers and possible mortality, adjusting for potential confounders. The optimal knots were selected by AIC, BIC, Harrell’s C-index, categorical net reclassification improvement (NRI) for events, continuous NRI for events and non-events, Smaller AIC and BIC values indicate a better the model fit. Harrell’s C-index measures the accuracy of the model’s prediction of the probability of event occurrence, with higher values indicating higher predictive accuracy. The NRI measures the degree of improvement in patient classification prediction by the model, including different types such as classification NRI, continuous NRI, event NRI, and non-event NRI. When classification NRI and continuous NRI increased, indicating that additional indicators may better help the model classify patients. Integrated discrimination improvement (IDI) is a metric for measuring the overall improvement of the model after adding new indicators, with larger values indicating greater overall improvement.

## Results

### Participant characteristics

This cohort study comprised 1,783 adults aged 18 years and older, including 929 males (52.10%), with a mean (SD) age of 62.49 (14.21) years; 499 participants (27.99%) were of Mexican American ethnicity, and 714 (40.04%) had non-Hispanic White ancestry (Table 1). The majority of the participants reported never engaging in physical activity (59.82%), with a slightly higher proportion of participants reporting vigorous activity than moderate activity. The duration of diabetes was < 3 years for 41.44% of the participants, 3–10 years for 28.24%, and > 10 years for 30.32%. The majority of participants used oral diabetes medication only (45.63%), with 18.58% using any insulin. Most participants had hypertension (71.83%), and more than half had hypercholesterolemia (53.29%). CVD event was present in 27.89% of participants. The medians of eGFR, CRP and hs-cTnT level were 83.32 (IQR, 63.08–97.41) mL/min per 1.73 m^2^, 3.60 (IQR, 1.60–7.60) mg/L and 9.92 (IQR, 6.12–16.62) ng/L, respectively, with no significant difference between the three time periods. Levels of cardiac biomarkers (hs-cTnT and hs-cTnI) and heart failure marker (NT-proBNP), which may indicate a higher risk of myocardial injury and heart failure in later stages. Levels of blood glucose and HbA1c gradually decreased during the study period, which may indicate an improvement in diabetes management, while the level of glycated albumin did not show significant changes. The levels of insulin and C-peptide and renal function marker (β-2 microglobulin) differed between groups in different years, but overall trends did not show obvious changes (P > 0.05).

**Table 1:** Baseline characteristics of subjects with diabetes among the study cohort (1999–2004)

	Total	1999–2000	2001–2002	2003–2004
**Participants, No**	1783	543	613	627
**Age, years**	62.49 ± 14.21	62.89 ± 13.02	61.58 ± 14.93	63.02 ± 14.43
**Male (%)**	929 (52.10)	278 (51.20)	325 (53.02)	326 (51.99)
**BMI, kg/m** ^**2**^	31.24 ± 6.81	31.12 ± 6.72	31.46 ± 6.99	31.14 ± 6.70
**Race/ethnicity**				
Mexican American	499 (27.99)	174 (32.04)	152 (24.80)	173 (27.59)
Other	158 (8.86)	59 (10.87)	53 (8.65)	46 (7.34)
Non-Hispanic white	714 (40.04)	175 (32.23)	258 (42.09)	281 (44.82)
Non-Hispanic black	412 (23.11)	135 (24.86)	150 (24.47)	127 (20.26)
**Education levels**				
Less than high school	867 (48.74)	326 (60.26)	275 (44.93)	266 (42.49)
High school or equivalent	362 (20.35)	101 (18.67)	127 (20.75)	134 (21.41)
Greater than high school	550 (30.92)	114 (21.07)	210 (34.31)	226 (36.10)
**Physical activity**				
Never	1066 (59.82)	346 (63.72)	362 (59.05)	358 (57.19)
Moderate	356 (19.98)	94 (17.31)	112 (18.27)	150 (23.96)
Vigorous	360 (20.20)	103 (18.97)	139 (22.68)	118 (18.85)
**Serum cotinine**				
> 10 ng/mL	378 (21.80)	106 (20.50)	127 (21.24)	145 (23.42)
LOD − 10 ng/mL	1116 (64.36)	411 (79.50)	345 (57.69)	360 (58.16)
< LOD	240 (13.84)	0 (0.00)	126 (21.07)	114 (18.42)
**Drinking status**	989 (59.22)	292 (57.37)	332 (57.84)	365 (62.18)
**PIR**				
≤ 1	387 (23.99)	132 (28.57)	118 (20.96)	137 (23.30)
1.01–4.99	1046 (64.85)	297 (64.29)	371 (65.90)	378 (64.29)
≥ 5	180 (11.16)	33 (7.14)	74 (13.14)	73 (12.41)
**Duration of diabetes, y**				
< 3	738 (41.44)	224 (41.40)	270 (44.05)	244 (38.92)
3–10	503 (28.24)	145 (26.80)	171 (27.90)	187 (29.82)
> 10	540 (30.32)	172 (31.79)	172 (28.06)	196 (31.26)
**Diabetes medication**				
No	203 (11.53)	58 (10.68)	75 (12.23)	70 (11.59)
Oral medication only	803 (45.63)	246 (45.30)	260 (42.41)	297 (49.17)
Any insulin use	327 (18.58)	102 (18.78)	110 (17.94)	115 (19.04)
Others	427 (24.26)	137 (25.23)	168 (27.41)	122 (20.20)
**Hypertension**	1280 (71.83)	387 (71.27)	435 (70.96)	458 (73.16)
**Hypercholesterolemia**	947 (53.29)	269 (49.63)	306 (50.33)	372 (59.33)
**CVD**	488 (27.89)	143 (26.68)	149 (25.08)	196 (31.61)
**eGFR, mL/min per 1.73 m** ^**2**^	83.32 (63.08–97.41)	83.47 (64.35–97.24)	84.95 (64.36–99.86)	82.60 (61.33–95.81)
**CRP, mg/L**	3.60 (1.60–7.60)	3.80 (1.80–8.50)	3.60 (1.60–7.40)	3.30 (1.50–7.30)
**hs-cTnT, ng/L**	9.92 (6.12–16.62)	9.12 (5.34–15.87)	9.39 (6.03–16.32)	11.05 (7.07–18.20)
**hs-cTnI, ng/L**	3.10 (1.80–6.10)	3.10 (1.80–6.70)	3.00 (1.70–5.90)	3.20 (1.90–5.90)
**NT-proBNP, pg/ml**	82.48 (35.82–232.50)	81.35 (38.35–214.50)	76.06 (29.71–223.60)	93.79 (37.73–259.00)
**Creatinine, umol/L**	79.56 (66.72–97.24)	75.68 (66.72–93.59)	79.56 (61.88–97.24)	79.56 (70.72–97.24)
**Cystatin C, mg/L**	0.85 (0.72–1.05)	0.85 (0.73–1.03)	0.86 (0.72–1.06)	0.84 (0.72–1.06)
**β-2 microglobulin, mg/L**	2.23 (1.86–2.91)	2.17 (1.82–2.81)	2.25 (1.85–2.91)	2.28 (1.87–3.07)
**Plasma glucose, mg/dL**	144.30 (124.80-187.20)	152.30 (129.30-200.50)	137.90 (123.50-179.80)	143.50 (121.20-186.90)
**HbA1c, %**	7.00 (6.20–8.20)	7.40 (6.40–8.80)	6.80 (6.10–8.10)	6.90 (6.10–7.90)
**Glycated albumin, %**	17.54 (15.10-22.03)	18.80 (15.58–23.05)	17.31 (15.00-21.28)	17.20 (14.95–21.24)
**Insulin, uU/mL**	16.63 (9.51–29.22)	19.96 (12.14–30.31)	17.57 (10.64–31.76)	14.00 (7.37–25.16)
**C-peptide, nmol/L**	1.10 (0.78–1.47)	1.17 (0.84–1.61)	1.04 (0.73–1.38)	1.11 (0.80–1.49)

BMI: body mass index; PIR: poverty-income ratio; CVD: cardiovascular disease; CRP: C-reactive protein; eGFR: estimated glomerular filtration rate; hs-cTnT: high-sensitivity Troponin T; hs-cTnI: high-sensitivity Troponin I; NT-proBNP,:N-terminal pro-B-type natriuretic peptide

### Correlation analysis of serum biomarkers

Supplement Fig. 1 illustrates a heat map that depicts the intricate correlation patterns observed among hs-cTnT, hs-cTnI, NT-proBNP, creatinine, cystatin C, and β-2 microglobulin in the diabetic population. The correlation estimates ranged from near 0 to strong positive correlations (0.81). These correlations may be attributed to shared physiological pathways or underlying disease mechanisms related to diabetes. Moreover, common confounding factors such as age, gender, and co-morbidities could also contribute to these correlations. Overall, this heat map provides valuable insights into the interplay among various biomarkers in diabetic individuals.

### All-cause and cause-specific mortality

Next, we investigated the association between higher levels of cardiac and renal biomarkers and the risk of death during the 7.43 years follow-up among diabetes (Supplement Table 1). For instance, when the hs-cTnT level is less than 6.11 ng/L, the CVD mortality rate is 0.5%, 1.8%, 3.7%, and 7.1% at 5, 10, 15, and 20 years, respectively. However, when the hs-cTnT level is greater than 16.67 ng/L, the CVD mortality rate sharply increases to 17.0%, 31%, 48%, and 61.6% at the same time points. Similarly, the levels of other biomarkers are also positively correlated with the mortality rate. For example, when the NT-proBNP level is greater than 232.60 pg/mL, the all-cause mortality rate is 36.3%, 64.4%, 82.8%, and 95.5%.

There is also a positive correlation between high levels of creatinine, cystatin C, and β-2 microglobulin and higher rates of all-cause and cause-specific mortality. For diabetes individuals with a cystatin C level of less than 0.72 mg/L, the CVD mortality rate is 0.7% at 5 years, 3.9% at 10 years, 5.9% at 15 years, and 9.9% at 20 years. Furthermore, 108 participants with creatinine levels ≥ 97.24 μmol/L died, while only 16.5 participants with creatinine levels < 66.72 μmol/L died. The group with the lowest level of β-2 microglobulin levels exhibited a CVD mortality rate of 1.8 per 1000 person-years, whereas the group with the highest β-2 microglobulin levels experienced a significantly elevated mortality rate of 16.1 per 1000 person-years.

Notably, the table shows that as the biomarker levels increase, the cumulative diabetic death rate and deaths per 1000 person-years also increase for hs-cTnI, NT-proBNP, creatinine, cystatin C, and β-2 microglobulin. Additionally, for hs-cTnT levels less than 6.11 ng/L, the cumulative diabetes death rate is 0% at 5 years, 0.8% at 10 years, 2.5% at 15 years, and 4.4% at 20 years, with diabetic deaths per 1000 person-years of 41.0 (31.4–49.2).

### Associations of biomarkers with mortality risk in diabetic patients

**Fig. 2:**
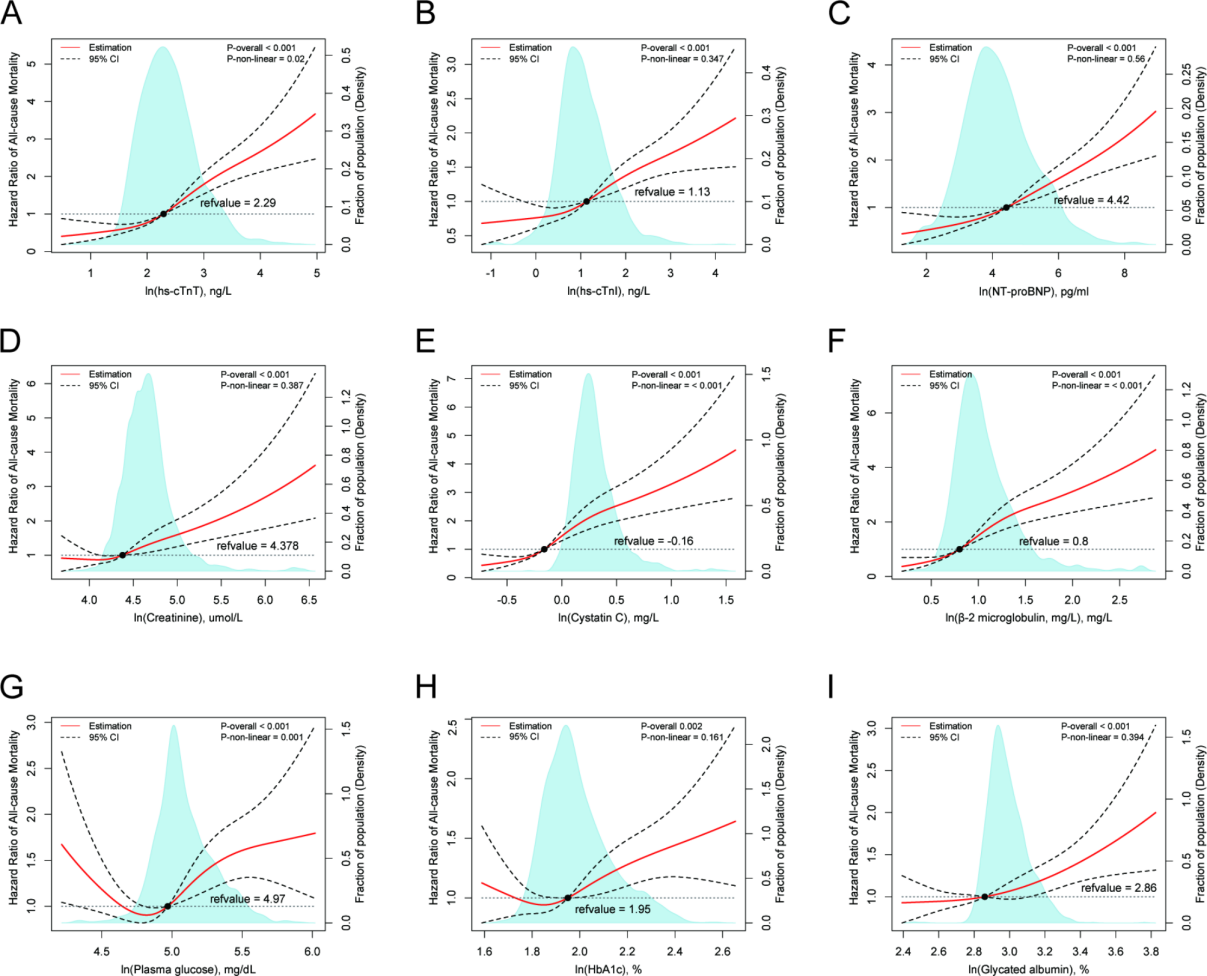
The nonlinear associations between cardio-renal and plasma glucose biomarkers with all-cause mortality in a diabetic population. The restricted cubic spline for the association between serum ln(hs-cTNT) (**A**), ln(hs-cTNI) (**B**), ln(NT-proBNP) (**C**), ln(Creatine) (**D**), ln(Cystatin C) (**E**), ln(β-2 microglobulin) (**F**), ln(plasma glucose) (**G**), ln(HbA1c) (**H**), ln(Glycated albumin) (**I**) and risk of all-cause mortality among adults with diabetes. Knots were placed at the 5th, 25th, 50th, and 75th percentiles of the serum biomarkers distribution. Adjustment factors were age, gender, education, marital status, race, the ratio of family income to poverty, BMI, drinking, smoking, physical activity. HR, hazard ratio; LL, lower limit; UL, upper limit

**Fig. 3:**
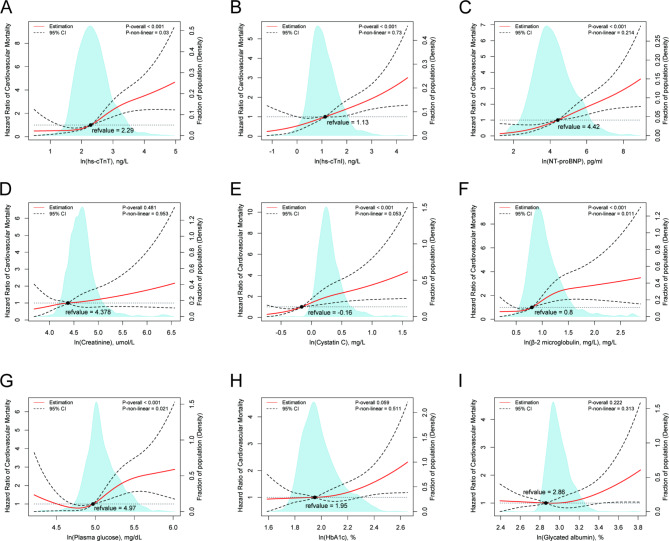
The nonlinear associations between cardio-renal and plasma glucose biomarkers with CVD mortality in a diabetic population. The restricted cubic spline for the association between serum ln(hs-cTNT) (**A**), ln(hs-cTNI) (**B**), ln(NT-proBNP) (**C**), ln(Creatine) (**D**), ln(Cystatin C) (**E**), ln(β-2 microglobulin) (**F**), ln(plasma glucose) (**G**), ln(HbA1c) (**H**), ln(Glycated albumin) (**I**) and risk of CVD mortality among adults with diabetes. Knots were placed at the 5th, 25th, 50th, and 75th percentiles of the serum biomarkers distribution. Adjustment factors were age, gender, education, marital status, race, the ratio of family income to poverty, BMI, drinking, smoking, physical activity. HR, hazard ratio; LL, lower limit; UL, upper limit

Specifically, hs-cTnT showed significant nonlinear associations with all-cause mortality (P-nonlinearity = 0.02, Fig. 2A) and CVD mortality risk (P-nonlinearity = 0.03, Fig. 3A), while cystatin C, β-2 microglobulin demonstrated significant nonlinear associations with all-cause mortality (P-nonlinearity < 0.001, Fig. 2E**&F**) and CVD mortality risk (P-nonlinearity = 0.011, Fig. 3F), respectively. Plasma glucose also exhibited a significant nonlinear association with all-cause mortality (P-nonlinearity = 0.001, Fig. 2G) and CVD mortality risk (P-nonlinearity = 0.021, Fig. 3G).

**Fig. 4:**
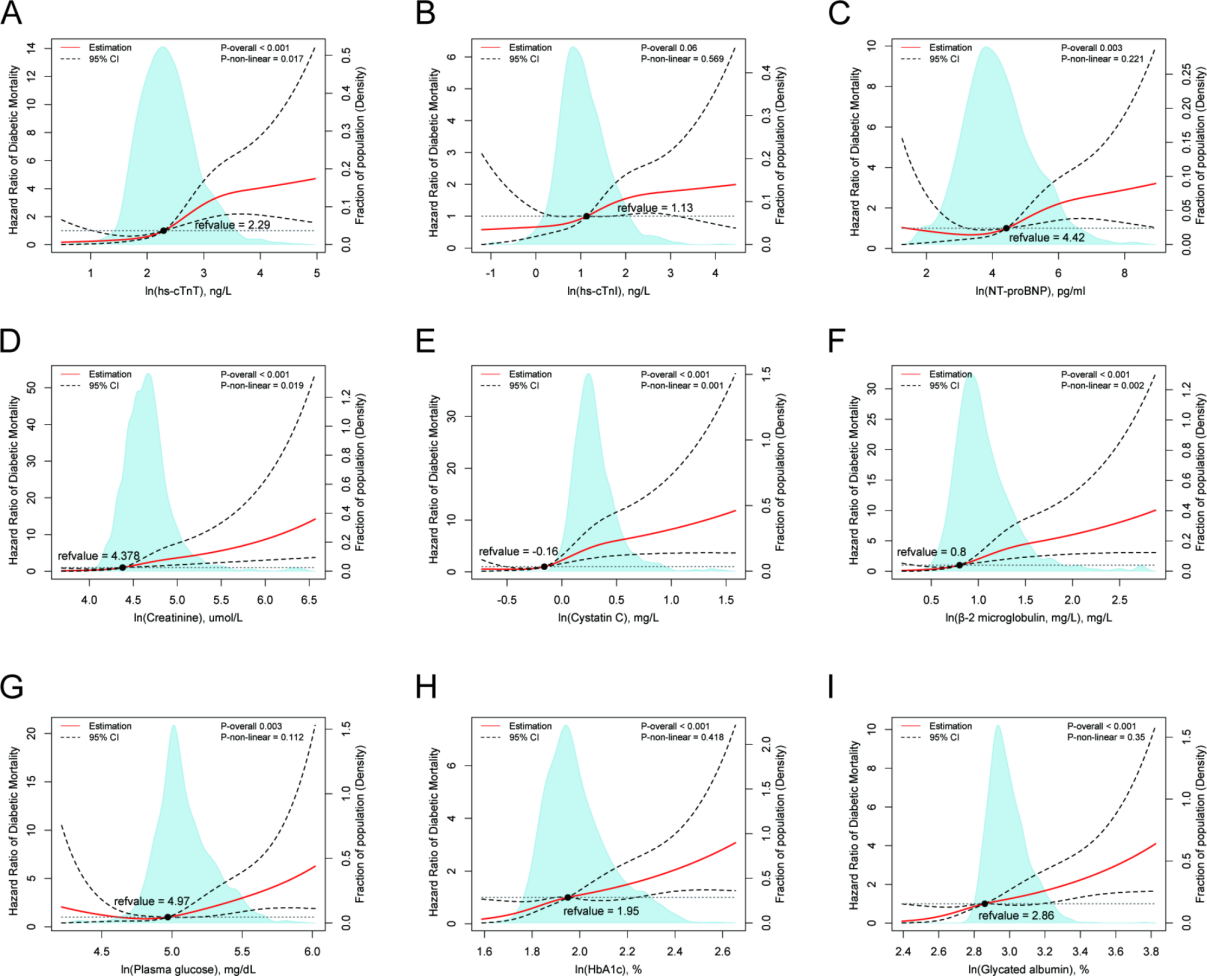
The nonlinear associations between cardio-renal and plasma glucose biomarkers with diabetic mortality in a diabetic population. The nonlinear associations between cardio-renal and plasma glucose biomarkers with diabetic mortality in a diabetic population. The restricted cubic spline for the association between serum ln(hs-cTNT) (**A**), ln(hs-cTNI) (**B**), ln(NT-proBNP) (**C**), ln(Creatine) (**D**), ln(Cystatin C) (**E**), ln(β-2 microglobulin) (**F**), ln(plasma glucose) (**G**), ln(HbA1c) (**H**), ln(Glycated albumin) (**I**) and risk of diabetic mortality among adults with diabetes. Knots were placed at the 5th, 25th, 50th, and 75th percentiles of the serum biomarkers distribution. Adjustment factors were age, gender, education, marital status, race, the ratio of family income to poverty, BMI, drinking, smoking, physical activity. HR, hazard ratio; LL, lower limit; UL, upper limit

Furthermore, hs-cTnT, creatinine, cystatin C, and β-2 microglobulin showed significant nonlinear associations with diabetic mortality risk in the same cohort (P-nonlinearity = 0.017, Fig. 4A; P-nonlinearity = 0.019, Fig. 4D; P-nonlinearity = 0.001, Fig. 4E; and P-nonlinearity = 0.002, Fig. 4F, respectively). However, no significant nonlinear association was observed between plasma glucose (P-nonlinearity = 0.112, Fig. 4G), HbA1c (P-nonlinearity = 0.418, Fig. 4H), and glycated albumin (P-nonlinearity = 0.35, Fig. 4I) and diabetic mortality risk in the same cohort.

Therefore, these findings indicate that the chosen biomarkers, such as hs-cTnT, hs-cTnI, NT-proBNP, creatinine, cystatin C, and β-2 microglobulin, may possess superior predictive value for mortality risk in diabetic patients when contrasted with glycemic control.

### Cox regression analysis on mortality

Table 2 presents the results of cox analysis to determine the relationship between serum biomarker levels and mortality rate, categorized by quartiles. For diabetes mortality, the probability of hs-cTnT, creatinine, and cystain C in the highest quartiles compared to the reference group were 6.32 times (95% CI: 3.09–17.35), 4.16 times (95% CI: 1.87–14.22), and 9.06 times (95% CI: 4.20-24.13) higher, respectively. However, for diabetes mortality, the conventional blood glucose monitoring indicators such as blood glucose levels, HbA1c, or glycated albumin showed only a modest increase in probability in the highest quartiles compared to the reference group, with a respective increase of 1.54 times (95% CI: 0.99–6.53), 2.19 times (95% CI: 1.65–6.19), and 5.36 times (95% CI: 2.62–15.47). This suggests that cardiac-renal biomarkers may be more sensitive in predicting mortality, particularly diabetes-related mortality.

**Table 2:** Association of Bio-markers Levels With All-Cause and Cause-Specific Mortality

	All-cause mortality			CVD mortality			Diabetes mortality		
	Unadjusted	Model 1	Model 2	Unadjusted	Model 1	Model 2	Unadjusted	Model 1	Model 2
**Variable**	HR (95% CI)	HR (95% CI)	HR (95% CI)	HR (95% CI)	HR (95% CI)	HR (95% CI)	HR (95% CI)	HR (95% CI)	HR (95% CI)
**hs-cTnT, ng/L**									
< 6.11	1	1	1	1	1	1	1	1	1
6.11-< 9.92	2.40 (1.88–3.07)	1.43 (1.10–1.86)	1.33 (1.00-1.76)	2.65 (1.56–4.50)	1.57 (0.91–2.73)	1.42 (0.79–2.58)	2.05 (0.98–4.32)	1.78 (0.82–3.88)	1.74 (0.76–3.97)
9.92-< 16.67	4.77 (3.77–6.03)	2.20 (1.69–2.86)	2.00 (1.50–2.65)	6.13 (3.74–10.06)	2.65 (1.54–4.56)	2.26 (1.26–4.03)	4.71 (2.37–9.35)	3.95 (1.85–8.44)	3.11 (1.35–7.14)
≥ 16.67	9.44 (7.50-11.88)	3.68 (2.80–4.84)	3.40 (2.52–4.60)	16.44 (10.21–26.48)	6.24 (3.61–10.81)	5.59 (3.08–10.15)	11.28 (5.82–21.86)	8.70 (4.00-18.91)	7.32 (3.09–17.35)
P trend	**< 0.001**	**< 0.001**	**< 0.001**	**< 0.001**	**< 0.001**	**< 0.001**	**< 0.001**	**< 0.001**	**< 0.001**
ln(hs-cTnT), ng/L	2.29 (2.14–2.45)	1.86 (1.69–2.04)	1.81 (1.62–2.03)	2.55 (2.27–2.87)	2.17 (1.85–2.56)	2.07 (1.69–2.54)	2.48 (2.06–2.99)	2.33 (1.84–2.94)	2.31 (1.71–3.12)
**hs-cTnI, ng/L**									
< 1.80	1	1	1	1	1	1	1	1	1
1.80-< 3.10	2.06 (1.64–2.58)	1.25 (0.99–1.58)	1.15 (0.90–1.48)	2.87 (1.72–4.77)	1.69 (1.01–2.84)	1.42 (0.83–2.43)	2.58 (1.37–4.88)	1.88 (0.98–3.62)	1.94 (0.94–3.99)
3.10-< 6.10	3.59 (2.90–4.46)	1.76 (1.39–2.23)	1.62 (1.25–2.10)	5.75 (3.55–9.32)	2.60 (1.56–4.34)	2.07 (1.20–3.55)	3.59 (1.91–6.75)	2.33 (1.19–4.55)	2.20 (1.02–4.76)
≥ 6.10	5.81 (4.70–7.18)	2.41 (1.89–3.06)	1.96 (1.50–2.56)	12.06 (7.57–19.22)	4.57 (2.74–7.61)	2.81 (1.63–4.86)	5.34 (2.86–9.97)	2.97 (1.48–5.96)	2.53 (1.14–5.61)
P trend	**< 0.001**	**< 0.001**	**< 0.001**	**< 0.001**	**< 0.001**	**< 0.001**	**< 0.001**	**0.002**	**0.036**
ln(hs-cTnI), ng/L	1.74 (1.65–1.84)	1.41 (1.31–1.52)	1.34 (1.23–1.46)	1.99 (1.82–2.19)	1.67 (1.48–1.89)	1.52 (1.30–1.77)	1.73 (1.47–2.03)	1.47 (1.20–1.80)	1.35 (1.06–1.72)
**NT-proBNP, pg/ml**									
< 35.77	1	1	1	1	1	1	1	1	1
35.77-< 82.82	1.75 (1.38–2.21)	1.28 (1.00-1.63)	1.20 (0.93–1.57)	2.24 (1.37–3.66)	1.71 (1.03–2.82)	1.54 (0.90–2.63)	1.72 (0.85–3.45)	1.33 (0.65–2.73)	1.23 (0.55–2.73)
82.82-< 232.60	3.44 (2.76–4.27)	1.88 (1.48–2.40)	1.73 (1.34–2.25)	3.96 (2.48–6.32)	2.37 (1.43–3.93)	2.15 (1.27–3.66)	3.85 (2.03–7.29)	2.38 (1.19–4.77)	2.39 (1.11–5.12)
≥ 232.60	7.18 (5.80–8.88)	2.98 (2.33–3.82)	2.46 (1.87–3.23)	12.87 (8.29–19.98)	5.89 (3.59–9.66)	3.93 (2.29–6.75)	7.96 (4.26–14.88)	4.09 (2.01–8.32)	3.67 (1.64–8.22)
P trend	**< 0.001**	**< 0.001**	**< 0.001**	**< 0.001**	**< 0.001**	**< 0.001**	**< 0.001**	**< 0.001**	**< 0.001**
ln(NT-proBNP), pg/ml	1.62 (1.56–1.69)	1.37 (1.30–1.44)	1.31 (1.23–1.39)	1.79 (1.67–1.91)	1.57 (1.43–1.71)	1.42 (1.28–1.59)	1.63 (1.45–1.83)	1.41 (1.22–1.63)	1.36 (1.15–1.63)
**Creatinine, umol/L**									
< 66.72	1	1	1	1	1	1	1	1	1
66.72-< 79.56	1.35 (1.11–1.63)	0.97 (0.79–1.19)	0.99 (0.79–1.24)	1.59 (1.11–2.28)	0.96 (0.65–1.42)	1.01 (0.66–1.54)	1.74 (0.96–3.15)	1.83 (0.98–3.43)	1.87 (0.94–3.74)
79.56-< 97.24	1.90 (1.59–2.28)	1.14 (0.93–1.41)	1.18 (0.92–1.51)	2.29 (1.63–3.23)	1.06 (0.71–1.57)	1.03 (0.65–1.63)	2.54 (1.44–4.46)	2.79 (1.49–5.24)	3.00 (1.41–6.40)
≥ 97.24	3.45 (2.89–4.12)	1.55 (1.25–1.94)	1.47 (1.04–2.09)	3.69 (2.62–5.21)	1.31 (0.86-2.00)	1.05 (0.55–2.03)	5.27 (3.06–9.08)	4.59 (2.40–8.77)	5.16 (1.87–14.22)
P trend	< 0.001	< 0.001	**0.038**	< 0.001	0.132	0.870	< 0.001	< 0.001	**0.001**
ln(Creatinine), umol/L	2.76 (2.45–3.10)	1.95 (1.65–2.30)	1.76 (1.38–2.24)	2.83 (2.29–3.50)	1.90 (1.37–2.62)	1.49 (0.92–2.41)	3.59 (2.69–4.80)	3.40 (2.41–4.80)	3.43 (2.04–5.79)
**Cystatin C, mg/L**									
< 0.72	1	1	1	1	1	1	1	1	1
0.72-< 0.85	2.33 (1.84–2.97)	1.62 (1.26–2.08)	1.68 (1.28–2.21)	2.23 (1.42–3.48)	1.43 (0.90–2.28)	1.56 (0.94–2.59)	2.32 (1.13–4.79)	2.20 (1.02–4.71)	1.90 (0.83–4.36)
0.85-< 1.05	3.65 (2.90–4.59)	1.81 (1.41–2.33)	1.92 (1.46–2.53)	3.48 (2.26–5.34)	1.47 (0.92–2.36)	1.68 (1.00-2.81)	3.22 (1.59–6.53)	2.55 (1.17–5.55)	2.61 (1.13-6.00)
≥ 1.05	8.45 (6.75–10.58)	3.36 (2.59–4.36)	3.54 (2.59–4.84)	9.02 (5.96–13.64)	3.25 (2.01–5.25)	3.26 (1.83–5.82)	11.82 (6.13–22.79)	8.15 (3.80-17.51)	10.06 (4.20-24.13)
P trend	**< 0.001**	**< 0.001**	**< 0.001**	**< 0.001**	**< 0.001**	**< 0.001**	**< 0.001**	**< 0.001**	**< 0.001**
ln(Cystatin C), mg/L	4.04 (3.59–4.55)	2.66 (2.26–3.14)	2.64 (2.11–3.31)	4.24 (3.43–5.25)	2.99 (2.22–4.02)	2.67 (1.74–4.08)	4.97 (3.65–6.77)	3.98 (2.72–5.82)	4.03 (2.33–6.97)
**β-2 microglobulin, mg/L**									
< 1.86	1	1	1	1	1	1	1	1	1
1.86-< 2.23	2.09 (1.65–2.65)	1.36 (1.06–1.74)	1.45 (1.10–1.90)	1.85 (1.19–2.86)	1.17 (0.74–1.86)	1.41 (0.85–2.35)	2.94 (1.35–6.38)	2.73 (1.20–6.23)	2.53 (1.03–6.21)
2.23-< 2.92	3.69 (2.95–4.61)	1.97 (1.55–2.51)	2.13 (1.63–2.77)	3.39 (2.25–5.09)	1.72 (1.10–2.68)	2.20 (1.35–3.59)	4.65 (2.20–9.84)	3.90 (1.72–8.87)	3.98 (1.64–9.64)
≥ 2.92	7.75 (6.23–9.64)	3.18 (2.48–4.07)	3.63 (2.69–4.89)	7.65 (5.16–11.36)	3.09 (1.97–4.86)	3.42 (1.97–5.93)	14.01 (6.86–28.61)	9.68 (4.27–21.92)	12.84 (5.04–32.70)
P trend	**< 0.001**	**< 0.001**	**< 0.001**	**< 0.001**	**< 0.001**	**< 0.001**	**< 0.001**	**< 0.001**	**< 0.001**
ln(β-2 microglobulin), mg/L	3.37 (3.04–3.73)	2.40 (2.09–2.75)	2.43 (2.01–2.94)	3.33 (2.76–4.03)	2.51 (1.94–3.23)	2.14 (1.48–3.10)	4.12 (3.18–5.36)	3.33 (2.41–4.62)	3.60 (2.26–5.72)
**Plasma glucose, mg/dL**									
< 124.70	1	1	1	1	1	1	1	1	1
124.70-< 144.30	0.93 (0.72–1.21)	0.94 (0.72–1.23)	0.99 (0.73–1.35)	0.98 (0.59–1.64)	1.01 (0.60–1.73)	1.33 (0.73–2.46)	0.67 (0.27–1.66)	0.65 (0.25–1.70)	0.63 (0.19–2.06)
144.30-< 188.00	1.13 (0.88–1.46)	1.15 (0.88–1.49)	1.18 (0.88–1.57)	1.57 (0.98–2.52)	1.60 (0.98–2.62)	1.93 (1.12–3.33)	1.64 (0.77–3.47)	1.93 (0.89–4.22)	1.66 (0.64–4.29)
≥ 188.00	1.24 (0.97–1.59)	1.56 (1.20–2.02)	1.67 (1.25–2.25)	1.86 (1.17–2.95)	2.45 (1.51–3.98)	3.33 (1.91–5.81)	1.63 (0.76–3.47)	2.12 (0.96–4.67)	2.54 (0.99–6.53)
P trend	0.033	< 0.001	< 0.001	0.001	< 0.001	< 0.001	0.056	0.009	0.011
ln(Plasma glucose), mmol/L	1.15 (0.91–1.46)	1.58 (1.21–2.05)	1.55 (1.15–2.08)	1.73 (1.15–2.60)	2.55 (1.61–4.05)	3.01 (1.80–5.03)	1.91 (0.97–3.75)	2.88 (1.37–6.07)	4.51 (1.84–11.08)
**HbA1c, %**									
< 6.20	1	1	1	1	1	1	1	1	1
6.20-< 7.00	0.92 (0.78–1.09)	0.89 (0.75–1.06)	0.93 (0.76–1.12)	1.10 (0.81–1.50)	1.11 (0.80–1.54)	1.09 (0.76–1.57)	1.29 (0.73–2.29)	1.22 (0.67–2.21)	1.41 (0.72–2.78)
7.00-< 8.20	0.93 (0.78–1.11)	1.08 (0.90–1.30)	1.05 (0.86–1.28)	1.02 (0.74–1.41)	1.29 (0.91–1.81)	1.21 (0.83–1.77)	1.94 (1.12–3.34)	2.19 (1.25–3.85)	2.29 (1.19–4.40)
≥ 8.20	0.83 (0.69–0.99)	1.28 (1.06–1.55)	1.35 (1.10–1.67)	0.93 (0.67–1.29)	1.51 (1.05–2.16)	1.47 (0.98–2.20)	1.89 (1.10–3.24)	2.90 (1.65–5.12)	3.19 (1.65–6.19)
P trend	0.047	0.003	0.004	0.557	0.017	0.056	0.007	< 0.001	< 0.001
ln(HbA1c), %	0.73 (0.56–0.97)	1.79 (1.31–2.44)	1.97 (1.39–2.79)	0.93 (0.56–1.53)	2.52 (1.43–4.44)	2.49 (1.31–4.73)	2.55 (1.25–5.19)	6.55 (2.99–14.38)	8.50 (3.43–21.06)
**Glycated albumin, %**									
< 15.09	1	1	1	1	1	1	1	1	1
15.09-< 17.54	1.27 (1.05–1.53)	0.94 (0.77–1.14)	0.91 (0.73–1.13)	1.41 (1.00-1.99)	0.97 (0.68–1.39)	0.95 (0.64–1.39)	3.78 (1.63–8.74)	3.56 (1.45–8.72)	2.78 (1.11–6.99)
17.54-< 22.10	1.34 (1.11–1.61)	1.13 (0.93–1.38)	1.06 (0.85–1.31)	1.29 (0.91–1.84)	1.03 (0.71–1.49)	0.85 (0.57–1.28)	5.98 (2.67–13.40)	6.44 (2.70-15.37)	4.97 (2.05–12.04)
≥ 22.10	1.28 (1.06–1.55)	1.41 (1.15–1.73)	1.43 (1.15–1.79)	1.38 (0.97–1.95)	1.50 (1.03–2.19)	1.44 (0.96–2.16)	6.67 (3.00-14.86)	8.76 (3.67–20.94)	6.36 (2.62–15.47)
P trend	0.008	< 0.001	0.001	0.130	0.038	0.173	< 0.001	< 0.001	< 0.001
ln(Glycated albumin), %	0.73 (0.56–0.97)	1.79 (1.31–2.44)	1.97 (1.39–2.79)	0.93 (0.56–1.53)	2.52 (1.43–4.44)	2.49 (1.31–4.73)	2.55 (1.25–5.19)	6.55 (2.99–14.38)	8.50 (3.43–21.06)

Hs-cTnT: high-sensitivity Troponin T; hs-cTnI: high-sensitivity Troponin I; NT-proBNP: N-terminal pro-B-type natriuretic peptide

Model 1: gender, age, race, BMI.

Model 2: gender, age, race, BMI, education, activity, PIR, cotinine, drinking, hypertension, hyperlipidemia, CVD, CKD.

### AIC, BIC, NRI and IDI for the combined assessment of biomarkers in predicting all-cause mortality

In Model 2, the incorporation of hs-cTnT, creatinine, cystatin C, and β-2 microglobulin yielded continuous NRI values of 0.564 (95% CI: 0.400-0.669), 0.188 (95% CI: 0.028–0.320), 0.357 (95% CI: 0.209–0.529), and 0.413 (95% CI: 0.261–0.559), respectively. The IDI values for these additions were 0.043 (95% CI: 0.024–0.058), 0.010 (95% CI: -0.001-0.021), 0.026 (95% CI: 0.006–0.038), and 0.031 (95% CI: 0.007–0.046), respectively. Conversely, when including blood glucose monitoring indicators in Model 2, the continuous NRI values were 0.107 (-0.057-0.330), 0.028 (-0.008-0.085), and 0.207 (-0.003-0.335), with corresponding IDI values of 0.002 (-0.006-0.017), 0.004 (-0.003-0.012), and 0.007 (-0.001-0.016).These results suggest that augmenting the model with cardiovascular and renal biomarkers from blood outperforms blood glucose monitoring indicators in predictive performance (Table 3). Similar results were observed for event NRI and non-event NRI. Importantly, when simultaneously introducing plasma hs-cTnT, NT-proBNP, creatinine, β-2 microglobulin, and glycated hemoglobin into Model 2, the IDI increased to 0.066. Compared to Model 1, Model 2 exhibited reduced AIC and BIC values, indicating that the inclusion of plasma hs-cTnT, NT-proBNP, creatinine, β-2 microglobulin, and glycated hemoglobin contributed to a better-fitting model.

**Table 3:** Comparison of Model Performance in Predicting All-Cause Mortality

Model	AIC	BIC	Harrell’s C-index	Categorical NRI	Continuous NRI	event NRI	nonevent NRI	IDI
Model 1	12620.09	12652.7	0.726 (0.71,0.742)	-	-	-	-	-
Model 1 + hs-cTnT	11107.78	11145.1	0.757 (0.741,0.773)	0.135 (0.072–0.205)	0.490 (0.338–0.616)	0.123 (0.032–0.198)	0.368 (0.282–0.456)	0.050 (0.030–0.064)
Model 1 + hs-cTnI	11163.33	11200.68	0.747 (0.731,0.763)	0.069 (0.016–0.133)	0.355 (0.271–0.488)	0.092 (0.019–0.170)	0.263 (0.198–0.353)	0.028 (0.014–0.044)
Model 1 + NT-proBNP	11165.26	11202.59	0.754 (0.738,0.771)	0.135 (0.082–0.194)	0.377 (0.265–0.519)	0.150 (0.053–0.232)	0.227 (0.172–0.317)	0.050 (0.025–0.071)
Model 1 + Creatinine	12193.66	12231.53	0.737 (0.721,0.753)	0.050 (-0.007-0.083)	0.191 (0.037–0.324)	-0.053 (-0.126-0.016)	0.243 (0.145–0.342)	0.020 (0.005–0.032)
**Model 1 + Cystatin C**	11209.89	11247.27	0.751 (0.735,0.767)	0.086 (0.010–0.146)	0.356 (0.194–0.519)	0.050 (-0.053-0.122)	**0.306 (0.202–0.445)**	0.040 (0.021–0.054)
**Model 1 + β-2 microglobulin**	11165.04	11202.4	0.754 (0.737,0.77)	0.106 (0.052–0.190)	0.425 (0.295–0.549)	0.052 (-0.026-0.140)	**0.373 (0.268–0.477)**	0.045 (0.021–0.061)
Model 1 + Plasma glucose	5651.761	5684.978	0.728 (0.706,0.75)	0.061 (-0.044-0.088)	0.110 (-0.038-0.282)	-0.103 (-0.221-0.000)	0.213 (0.129–0.303)	0.001 (-0.007-0.013)
Model 1 + HbA1c	12609.06	12647.11	0.729 (0.713,0.744)	0.053 (-0.028-0.066)	0.123 (0.010–0.277)	-0.087 (-0.165-0.010)	0.210 (0.156–0.288)	0.003 (-0.002-0.009)
Model 1 + Glycated albumin	8747.793	8850.299	0.734 (0.718,0.751)	0.026 (-0.004-0.072)	0.214 (0.094–0.324)	0.000 (-0.084-0.067)	0.214 (0.140–0.292)	0.006 (-0.001-0.014)
Model 2	10125.14	10224.76		-	-	-	-	-
**Model 2 + hs-cTnT**	**9249.943**	**9353.346**	0.746 (0.729,0.763)	0.106 (0.059–0.190)	**0.564 (0.400-0.669)**	**0.175 (0.063–0.225)**	**0.390 (0.298–0.474)**	**0.043 (0.024–0.058)**
Model 2 + hs-cTnI	9302.74	9406.189	0.765 (0.748,0.782)	0.046 (-0.005-0.096)	0.295 (0.148–0.437)	0.039 (-0.061-0.112)	0.256 (0.162–0.383)	0.019 (0.008–0.031)
Model 2 + NT-proBNP	9307.591	9411.009	0.757 (0.74,0.775)	0.086 (0.031–0.145)	0.381 (0.236–0.513)	0.158 (0.072–0.242)	0.222 (0.141–0.313)	0.032 (0.014–0.050)
**Model 2 + Creatinine**	**10108.76**	**10213.63**	0.76 (0.743,0.777)	0.032 (-0.012-0.070)	**0.188 (0.028–0.320)**	**-0.005 (-0.086-0.065)**	**0.192 (0.089–0.285)**	**0.010 (-0.001-0.021)**
**Model 2 + Cystatin C**	**9347.044**	**9450.569**	0.75 (0.732,0.767)	0.073 (0.013–0.149)	**0.357 (0.209–0.529)**	**0.022 (-0.060-0.132)**	**0.335 (0.197–0.436)**	**0.026 (0.006–0.038)**
**Model 2 + β-2 microglobulin**	**9307.106**	**9410.585**	0.76 (0.743,0.777)	0.107 (0.023–0.143)	**0.413 (0.261–0.559)**	**0.053 (-0.034-0.149)**	**0.360 (0.271–0.485)**	**0.031 (0.007–0.046)**
Model 2 + Plasma glucose	4574.251	4665.387	0.762 (0.745,0.78)	0.009 (-0.034-0.084)	0.107 (-0.057-0.330)	-0.080 (-0.163-0.079)	0.188 (0.054-0.300)	0.002 (-0.006-0.017)
Model 2 + HbA1c	10113.25	10218.12	0.758 (0.740–0.775)	0.014 (-0.014-0.062)	0.028 (-0.008-0.085)	0.021 (-0.022-0.063)	0.007 (-0.006-0.027)	0.004 (-0.003-0.012)
Model 2 + Glycated albumin	8697.23	8799.687	0.760 (0.742–0.777)	0.231 (0.088–0.346)	0.207 (-0.003-0.335)	0.000 (-0.140-0.071)	0.207 (0.118–0.303)	0.007 (-0.001-0.016)

Hs-cTnT: high-sensitivity Troponin T; hs-cTnI: high-sensitivity Troponin I; NT-proBNP: N-terminal pro-B-type natriuretic peptid

Model 1: gender, age, race, BMI.

Model 2: gender, age, race, BMI, education, activity, PIR, cotinine, drinking, hypertension, hyperlipidemia, CVD, CKD.

## Discussion

The prevalence of type 2 diabetes and the burden of its complications are progressively escalating. Early diagnosis and management of diabetes are crucial to prevent the development of long-term complications such as cardiovascular disease, kidney failure, and nerve damage [12]. Diabetic patients are at a higher and earlier risk of developing CVD, with a 2–4 times increased risk of mortality compared to non-diabetic patients [13]. Blood glucose, HbA1c, and glycated albumin are commonly used indicators for diagnosing diabetes or monitoring blood glucose control [14]. However, each indicator has limitations [15]. Blood glucose levels can be influenced by many factors, making a single measurement of blood glucose inaccurate. Additionally, some individuals may show pre-diabetes in an oral glucose tolerance test but have normal blood glucose levels in daily life, making it challenging to diagnose diabetes accurately. HbA1c measurement reflects the average blood glucose level over the past 2–3 months but cannot provide information on blood glucose fluctuations over a shorter period. Furthermore, some factors, such as iron-deficiency anemia, liver disease, and severe anemia, may affect the accuracy of HbA1c measurement [16]. Previous research has unequivocally demonstrated the significance of kidney function and albumin uria as major cardiovascular risk factors. In tandem with the rise of personalized medicine, we find ourselves at the threshold of a more refined and bespoke era in healthcare management. Our fervent pursuit centers on a comprehensive exploration of the profound influence wielded by biomarkers on individual health profiles, with a particular emphasis on the identification of cardiac and renal biomarkers-a task of paramount significance for healthcare professionals. This process of identification is poised to facilitate the pinnacle of precision in healthcare, as it empowers us to precisely pinpoint those diabetic patients most in need of vigilance and intervention, thereby precipitating a reduction in the incidence of cardiovascular eventss [17]. As shown in Supplement Table 1, the adjusted Model 2 demonstrates a significant association of cardiac-renal biomarkers with all-cause, CVD and diabetes mortality (P trend < 0.05). However, HbA1C and glycated albumin did not exhibit a significant association with CVD mortality(P trend > 0.05).

Table 2 provides a comprehensive evaluation of two models in predicting death events based on various biomarkers. As expected, both all-cause and cause-specific mortality significantly increased over time and with increasing values of hs-cTnT and hs-cTnI within their highest quartile, suggesting that these markers may be used to assess the risk of cardiovascular disease and diabetes, leading to more serious health consequences [18]. Hs-cTnT and hs-cTnI are markers of myocardial injury, and their elevation in diabetic patients is closely associated with an increased risk of cardiovascular disease and death [18, 19]. NT-proBNP is a marker of heart failure and increases when myocardial cells are damaged or when the heart is under increased load, making it a predictive marker for cardiovascular death. Within the range of NT-proBNP values, the mortality rate for CVD is higher than that for diabetes, further indicating a more significant impact of cardiovascular disease on patient health [20, 21]. In Model 2, the incorporation of cardiac and renal biomarkers, namely hs-cTnT, creatinine, cystatin C, and β-2 microglobulin, resulted in a notable enhancement in predictive performance. This improvement was evident in both continuous NRI and IDI, signifying a significant boost in the model’s predictive accuracy. Importantly, our findings highlight the superiority of cardiac and renal biomarkers over blood glucose monitoring indicators. Additionally, the simultaneous inclusion of plasma biomarkers, specifically hs-cTnT, NT-proBNP, creatinine, β-2 microglobulin, and glycated hemoglobin, further elevated the IDI, indicating a substantial enhancement in the model’s predictive capacity. In summary, our study provides essential insights for leveraging these biomarkers in the contemporary era of more precise diagnostics and broader availability of anti-diabetic medications, facilitating improved assessment of long-term mortality risk associated with diabetes (Table 3).

Creatinine is a product of muscle metabolism and an indicator of kidney function that is more commonly used. Changes in creatinine concentration can indicate changes in kidney filtration function [22]. Beta-2 microglobulin is an indicator of renal tubular function that is frequently used to assess kidney disease and the effectiveness of kidney transplants [23]. In the case of renal tubular injury, the concentration of beta-2 microglobulin increases, making it a useful marker for kidney disease. Compared to creatinine and cystatin C, beta-2 microglobulin is more sensitive in detecting early damage to renal tubular function [24]. To minimize the influence of confounding diseases such as heart failure and renal failure on these cardio-renal biomarkers, we employed a multivariate regression model that incorporated potential influencing factors. We evaluated the accuracy and predictive capacity of the model by measuring AIC, BIC, IDI, and other indicators. The inclusion of these variables significantly increased the reliability of the results. The newer Risk Equations for Complications Of Type 2 Diabetes (RECODe) model, designed for predicting 10-year risks, was derived from clinical trials conducted in the United States and Canada and subsequently validated in North American trial settings and cohort studies. In our investigation, we juxtaposed our Model 2 against the established RECODe model. Overall, we observed negligible discrepancies in the alterations of key indicators, including AIC, BIC, and IDI, which implies that our Model 2 exhibits a commendable level of reliability (Supplement Table 2).

It is noteworthy that clinical guidelines concerning hypertension and cardiovascular disease only provide limited mention of kidney markers. Additionally, crucial risk factors, such as microalbuminuria, cystatin C, and cTNI/cTNT have not been incorporated into commonly employed risk equations for evaluating cardiovascular complications in individuals with diabetes. Consequently, we strongly advocate for intensified collaboration between nephrologists and cardiologists in research, guideline development, and clinical practice to integrate the optimal cutoff values we have identified for predicting mortality risk in patients with diabetes. For example, elevated creatinine levels in patients indicate a need for strengthening renal function monitoring and interventions, along with controlling blood pressure and glucose levels, to prevent further progression to renal failure. Similarly, elevated NT-proBNP levels in patients indicate a need for strengthening cardiovascular monitoring and effective treatments to prevent heart failure. Given the high prevalence of diabetes and its associated complications, identifying patients at risk of adverse outcomes is critical for preventing the progression of disease and reducing the risk of mortality. The investigation of alternative blood markers related to cardiovascular and renal function could provide more insights into the long-term prognosis of diabetes patients beyond traditional glycemic markers such as HbA1c and fasting blood glucose levels.

Overall, this study underscores the significance of biomarker monitoring in clinical practice and emphasizes the importance of personalized interventions for improving patient outcomes. By leveraging these biomarkers, physicians can identify high-risk patients, tailor their treatments accordingly, and ultimately improve the quality of care provided to patients.

### Limitations

Although the NHANES study has a large sample size, it may be affected by selection bias and limitations in sample distribution and sampling methods, which could impact result validity. Laboratory errors could also influence blood biomarker measurements, reducing prediction model accuracy. The selected biomarkers may not fully capture underlying biological mechanisms, especially for long-term mortality prediction. Additional biomarkers may be necessary to improve the model’s accuracy. Other health risk factors, such as genetics, lifestyle, and disease history, may also affect biomarker interpretation. Despite these limitations, our study offers a breakthrough in predicting 20-year mortality risk by exploring new blood biomarkers beyond traditional glucose biomarkers. Further research is needed to fully understand their clinical value.

## Conclusions

Cardiovascular and renal biomarkers offer a promising approach to improve the prediction of outcomes in diabetic patients. These biomarkers, with their ability to reflect early disease activity, high sensitivity, and specificity, show potential in enhancing our ability to predict diabetes patients prognosis.

### Electronic supplementary material

Below is the link to the electronic supplementary material.

**Supplementary Material 1: Supplement Table 1**. All-cause and Cause-Specific Mortality rates per 1000 person years [95% Confidence Interval (CI)]. **Supplement Table 2**. Comparison of Model Performance in Predicting All-Cause Mortality. **Supplement Figure 1**. A heat map shows the correlations among different biomarkers including hs-cTnT, hs-cTnI, NT-proBNP, creatinine, cystatin C, and β-2 microglobulin in a population of diabetic patients. The strength of the correlations ranged from near 0 to 0.81, indicating complex interrelationships that may arise from shared physiological pathways, underlying disease mechanisms related to diabetes, or common confounding factors such as age, gender, and co-morbidities. These findings highlight the need for a comprehensive understanding of the relationships among biomarkers in diabetic individuals for effective management and care.

## Data Availability

The datasets generated and/or analyzed during the current study are publicly available from the website (https://wwwn.cdc.gov/nchs/nhanes/continuousnhanes/default.aspx?BeginYear=1999).

## Abbreviations

AIC: Akaike information criterion
BIC: Bayesian information criterion
CIs: Confidence intervals
CKD: Chronic kidney disease
CVD: Cardiovascular disease
eGFR: Estimated glomerular filtration rate
HRs: Hazard ratios
hs-cTnT: High-sensitivity cardiac troponin T
IDI: Integrated discrimination improvement
KM: Kaplan-meier
NDI: National death index
NRI: Net reclassification improvement
RCS: Restricted cubic spline
SDs: Standard deviations
