# Supplementary material for: Association of cardio-renal biomarkers and mortality in the U.S.: a prospective cohort study
Source: Cardiovasc Diabetol. 2023 Sep 29;22:265. doi: 10.1186/s12933-023-01986-2 (PMC10542251; doi:10.1186/s12933-023-01986-2)

**Supplement Table 1. All-cause and Cause-Specific Mortality rates per 1000 person years [95% Confidence Interval (CI)]**

|  | **5 years** | | | **10 years** | | | **15 years** | | | **20 years** | | | **Deaths/ 1000 person-yrs** | | |
| --- | --- | --- | --- | --- | --- | --- | --- | --- | --- | --- | --- | --- | --- | --- | --- |
| **Variable** | All-cause  mortality | CVD  mortality | Diabetes  mortality | All-cause  mortality | CVD  mortality | Diabetes  mortality | All-cause  mortality | CVD  mortality | Diabetes  mortality | All-cause  mortality | CVD  mortality | Diabetes   mortality | All-cause  mortality | CVD  mortality | Diabetes  mortality |
| **hs-cTnT, ng/L** |  |  |  |  |  |  |  |  |  |  |  |  |  |  |  |
| < 6.11 | 2.2% | 0.5% | 0.0% | 8.2% | 1.8% | 0.8% | 16.9% | 3.7% | 2.5% | 29.2% | 7.1% | 4.4% | 14.3(11.7-17.5) | 27.6(24.0-31.6) | 41.0(34.1-49.2) |
| 6.11-< 9.92 | 6.7% | 1.3% | 0.0% | 20.4% | 5.5% | 0.8% | 36.1% | 10.9% | 4.6% | 54.9% | 14.6% | 7.8% | 32.6(28.2-37.5) | 36.1(31.6-41.2) | 38.5(32.1-46.2) |
| 9.92-< 16.67 | 12.9% | 3.1% | 1.6% | 39.8% | 10.9% | 7.1% | 60.5% | 22.3% | 9.1% | 82.2% | 38.2% | 12.8% | 60.0(53.4-67.4) | 50.3(44.6-56.7) | 46.4(39.1-55.0) |
| ≥ 16.67 | 38.8% | 17.0% | 6.2% | 64.2% | 31.0% | 13.2% | 82.8% | 48.0% | 21.5% | 92.0% | 61.6% | 26.4% | 108.0(97.3-119.8) | 86.3(77.1-96.6) | 50.4(42.7-59.6) |
| **hs-cTnI, ng/L** |  |  |  |  |  |  |  |  |  |  |  |  |  |  |  |
| < 1.80 | 4.7% | 0.7% | 0.0% | 12.1% | 1.0% | 1.5% | 20.8% | 3.5% | 2.7% | 32.0% | 6.8% | 5.5% | 3.0(1.9-4.7) | 7.2(5.5-9.4) | 10.0(6.9-14.4) |
| 1.80-< 3.10 | 6.7% | 1.3% | 1.3% | 20.7% | 5.5% | 3.3% | 40.2% | 12.3% | 7.8% | 60.2% | 21.8% | 11.0% | 7.6(5.6-10.2) | 11.2(8.9-14.3) | 9.9(6.9-14.1) |
| 3.10-< 6.10 | 16.9% | 4.1% | 2.0% | 40.2% | 12.9% | 7.0% | 59.1% | 23.3% | 10.2% | 76.3% | 31.1% | 12.7% | 16.3(13.0-20.3) | 15.9(12.9-19.7) | 15.6(11.6-20.9) |
| ≥ 6.10 | 32.6% | 15.1% | 4.0% | 59.1% | 29.3% | 8.4% | 77.8% | 41.5% | 14.4% | 86.5% | 48.1% | 17.0% | 39.6(33.3-47.0) | 24.4(19.7-30.1) | 18.4(14.0-24.3) |
| **NT-proBNP, pg/mL** |  |  |  |  |  |  |  |  |  |  |  |  |  |  |  |
| < 35.77 | 3.7% | 0.5% | 0.2% | 10.9% | 1.6% | 1.6% | 20.8% | 4.1% | 2.7% | 33.8% | 10.2% | 4.4% | 1.7(0.9-3.0) | 2.5(1.6-4.0) | 3.9(2.2-7.0) |
| 35.77-< 82.82 | 6.5% | 1.3% | 0.5% | 19.7% | 5.1% | 1.7% | 34.6% | 10.8% | 4.4% | 50.8% | 16.7% | 8.5% | 3.3(2.1-5.1) | 4.4(3.0-6.4) | 2.6(1.3-5.2) |
| 82.82-< 232.60 | 13.7% | 3.5% | 0.8% | 37.2% | 10.0% | 5.8% | 58.3% | 20.4% | 11.4% | 80.5% | 28.1% | 14.5% | 7.0(5.0-9.8) | 6.2(4.4-8.7) | 6.4(4.0-10.1) |
| ≥ 232.60 | 36.3% | 16.1% | 6.2% | 64.4% | 32.4% | 13.0% | 82.8% | 47.7% | 18.3% | 95.5% | 58.7% | 20.9% | 14.9(11.3-19.7) | 12.0(8.9-16.3) | 6.3(3.9-10.0) |
| **Creatinine, umol/L** |  |  |  |  |  |  |  |  |  |  |  |  |  |  |  |
| < 66.72 | 7.2% | 0.6% | 0.6% | 18.3% | 4.0% | 2.5% | 33.1% | 10.5% | 3.7% | 48.0% | 15.3% | 6.1% | 17.8(14.9-21.3) | 16.5(13.6-20.1) | 48.4(43.0-54.5) |
| 66.72-< 79.56 | 9.6% | 4.2% | 0.7% | 25.6% | 10.2% | 2.4% | 42.1% | 15.1% | 7.1% | 60.6% | 25.1% | 8.3% | 35.5(31.0-40.8) | 35.1(30.6-40.3) | 44.9(39.9-50.6) |
| 79.56-< 97.24 | 13.2% | 5.4% | 0.8% | 35.0% | 12.7% | 4.2% | 53.3% | 21.6% | 9.3% | 68.2% | 29.8% | 13.0% | 59.2(52.6-66.7) | 52.7(46.8-59.4) | 45.4(40.0-51.4) |
| ≥ 97.24 | 33.3% | 11.3% | 5.8% | 56.7% | 21.2% | 13.2% | 73.1% | 32.1% | 15.1% | 84.4% | 36.8% | 20.3% | 90.6(81.3-101.0) | 108.0(97.4-119.9) | 40.7(35.8-46.2) |
| **Cystatin C, mg/L** |  |  |  |  |  |  |  |  |  |  |  |  |  |  |  |
| < 0.72 | 4.2% | 0.7% | 0.0% | 12.0% | 3.9% | 1.1% | 20.2% | 5.9% | 2.2% | 28.1% | 9.9% | 3.5% | 3.1(2.1-4.8) | 4.8(3.3-6.9) | 13.2(10.5-16.5) |
| 0.72-< 0.85 | 9.9% | 2.3% | 0.8% | 21.9% | 6.0% | 2.0% | 38.7% | 13.7% | 5.4% | 58.2% | 20.1% | 9.8% | 8.8(6.6-11.5) | 9.7(7.5-12.6) | 14.5(11.8-17.9) |
| 0.85-< 1.05 | 11.8% | 4.6% | 0.8% | 34.6% | 9.6% | 4.5% | 54.3% | 20.0% | 8.5% | 74.5% | 31.3% | 10.8% | 16.8(13.4-20.9) | 14.6(11.7-18.3) | 13.5(10.7-17.0) |
| ≥ 1.05 | 35.3% | 13.7% | 6.1% | 64.4% | 29.4% | 14.9% | 84.0% | 42.2% | 22.6% | 92.2% | 48.9% | 28.0% | 33.4(27.9-39.9) | 33.8(28.1-40.7) | 12.4(9.8-15.6) |
| **β-2 microglobulin, mg/L** |  |  |  |  |  |  |  |  |  |  |  |  |  |  |  |
| < 1.86 | 4.6% | 0.7% | 0.0% | 11.7% | 4.4% | 1.1% | 19.7% | 6.3% | 1.9% | 32.2% | 10.8% | 4.1% | 2.1(1.2-3.5) | 1.8(1.0-3.3) | 3.6(2.3-5.5) |
| 1.86-< 2.23 | 9.1% | 3.1% | 0.5% | 22.4% | 6.3% | 2.0% | 38.2% | 12.5% | 5.9% | 54.7% | 19.8% | 7.8% | 5.3(3.7-7.5) | 3.8(2.5-5.8) | 4.6(3.2-6.7) |
| 2.23-< 2.92 | 11.2% | 3.6% | 1.1% | 34.3% | 9.5% | 4.5% | 56.0% | 22.7% | 8.0% | 76.3% | 32.7% | 12.1% | 7.0(4.9-9.8) | 5.1(3.5-7.4) | 6.9(5.0-9.6) |
| ≥ 2.92 | 35.6% | 13.5% | 6.1% | 64.1% | 28.1% | 14.9% | 82.7% | 39.6% | 22.3% | 91.6% | 47.2% | 27.8% | 9.7(7.0-13.5) | 16.1(12.3-21.1) | 6.8(5.0-9.3) |
| **Plasma glucose, mg/dL** |  |  |  |  |  |  |  |  |  |  |  |  |  |  |  |
| < 124.70 | 16.7% | 4.8% | 2.0% | 35.1% | 9.7% | 3.2% | 46.0% | 14.4% | 5.3% | 56.7% | 16.6% | 7.5% | 17.9(14.9-21.5) | 16.4(13.6-19.8) | 37.2(32.4-42.7) |
| 124.70-< 144.30 | 12.6% | 4.8% | 0.5% | 26.0% | 7.3% | 1.6% | 44.0% | 13.5% | 3.6% | 59.3% | 19.6% | 5.5% | 30.6(26.5-35.4) | 33.1(28.7-38.2) | 46.5(41.0-52.7) |
| 144.30-< 188.00 | 13.1% | 4.7% | 1.0% | 32.6% | 12.8% | 4.5% | 52.0% | 21.7% | 10.2% | 62.1% | 29.1% | 12.1% | 56.4(50.1-63.4) | 55.3(49.1-62.2) | 49.1(43.4-55.7) |
| ≥ 188.00 | 17.7% | 6.2% | 2.5% | 34.8% | 13.6% | 5.5% | 54.3% | 25.2% | 8.5% | 68.7% | 33.9% | 16.4% | 106.3(95.8-118.0) | 105.5(95.1-117.1) | 47.2(41.5-53.6) |
| **HbA1c, %** |  |  |  |  |  |  |  |  |  |  |  |  |  |  |  |
| < 6.20 | 18.1% | 6.3% | 1.7% | 35.7% | 11.4% | 2.9% | 52.1% | 18.5% | 4.4% | 63.8% | 22.7% | 7.4% | 3.7(2.5-5.6) | 5.0(3.6-7.0) | 10.5(8.1-13.6) |
| 6.20-< 7.00 | 13.7% | 5.3% | 1.2% | 32.5% | 13.4% | 3.8% | 49.6% | 20.4% | 7.1% | 63.7% | 24.9% | 8.7% | 8.2(6.2-10.8) | 9.0(6.8-11.8) | 14.7(11.7-18.3) |
| 7.00-< 8.20 | 13.4% | 3.8% | 1.6% | 32.9% | 10.1% | 6.4% | 48.9% | 17.4% | 10.1% | 65.3% | 27.6% | 12.7% | 13.5(10.6-17.2) | 15.7(12.6-19.6) | 13.4(10.5-17.0) |
| ≥ 8.20 | 15.7% | 4.9% | 2.5% | 30.3% | 9.9% | 6.0% | 45.3% | 17.8% | 10.4% | 61.3% | 24.0% | 14.9% | 39.6(33.4-47.0) | 32.2(26.7-38.9) | 14.3(11.3-18.0) |
| **Glycated albumin, %** |  |  |  |  |  |  |  |  |  |  |  |  |  |  |  |
| < 15.09 | 12.6% | 4.4% | 0.5% | 27.7% | 9.2% | 0.8% | 42.3% | 15.3% | 1.6% | 56.9% | 17.9% | 2.8% | 2.0(1.2-3.5) | 1.4(0.7-2.6) | 1.3(0.6-2.7) |
| 15.09-< 17.54 | 12.4% | 5.1% | 1.1% | 31.2% | 11.6% | 3.9% | 51.0% | 19.2% | 7.1% | 65.2% | 27.4% | 9.3% | 3.4(2.2-5.3) | 3.9(2.6-5.9) | 4.8(3.3-7.1) |
| 17.54-< 22.10 | 16.8% | 4.3% | 2.4% | 35.4% | 10.5% | 6.6% | 52.0% | 18.8% | 10.5% | 66.9% | 26.1% | 16.0% | 7.2(5.2-10.0) | 5.8(4.1-8.4) | 7.6(5.5-10.4) |
| ≥ 22.10 | 18.4% | 6.4% | 3.0% | 37.2% | 13.1% | 7.9% | 50.4% | 19.6% | 12.7% | 65.0% | 28.6% | 15.5% | 13.2(9.8-17.7) | 15.5(11.8-20.3) | 8.5(6.3-11.4) |

Hs-cTnT: high-sensitivity Troponin T; hs-cTnI: high-sensitivity Troponin I; NT-proBNP: N-terminal pro-B-type natriuretic peptide.

**Supplement Table 2. Comparison of Model Performance in Predicting All-Cause Mortality**

| Model | AIC | BIC | Harrell’s C-index | Categorical NRI | Continuous NRI | event NRI | nonevent NRI | IDI |
| --- | --- | --- | --- | --- | --- | --- | --- | --- |
| Model 1 | 12620.09 | 12652.7 | 0.726(0.71,0.742) | - | - | - | - | - |
| Model 1 + hs-cTnT | 11107.78 | 11145.1 | 0.757(0.741,0.773) | 0.135(0.072-0.205) | 0.490(0.338-0.616) | 0.123(0.032-0.198) | 0.368(0.282-0.456) | 0.050(0.030-0.064) |
| Model 1 + hs-cTnI | 11163.33 | 11200.68 | 0.747(0.731,0.763) | 0.069(0.016-0.133) | 0.355(0.271-0.488) | 0.092(0.019-0.170) | 0.263(0.198-0.353) | 0.028(0.014-0.044) |
| Model 1 + NT-proBNP | 11165.26 | 11202.59 | 0.754(0.738,0.771) | 0.135(0.082-0.194) | 0.377(0.265-0.519) | 0.150(0.053-0.232) | 0.227(0.172-0.317) | 0.050(0.025-0.071) |
| Model 1 + Creatinine | 12193.66 | 12231.53 | 0.737(0.721,0.753) | 0.050(-0.007-0.083) | 0.191(0.037-0.324) | -0.053(-0.126-0.016) | 0.243(0.145-0.342) | 0.020(0.005-0.032) |
| **Model 1 + Cystatin C** | 11209.89 | 11247.27 | 0.751(0.735,0.767) | 0.086(0.010-0.146) | 0.356(0.194-0.519) | 0.050(-0.053-0.122) | **0.306(0.202-0.445)** | 0.040(0.021-0.054) |
| **Model 1 + β-2 microglobulin** | 11165.04 | 11202.4 | 0.754(0.737,0.77) | 0.106(0.052-0.190) | 0.425(0.295-0.549) | 0.052(-0.026-0.140) | **0.373(0.268-0.477)** | 0.045(0.021-0.061) |
| Model 1 + Plasma glucose | 5651.761 | 5684.978 | 0.728(0.706,0.75) | 0.061(-0.044-0.088) | 0.110(-0.038-0.282) | -0.103(-0.221-0.000) | 0.213(0.129-0.303) | 0.001(-0.007-0.013) |
| Model 1 + HbA1c | 12609.06 | 12647.11 | 0.729(0.713,0.744) | 0.053(-0.028-0.066) | 0.123(0.010-0.277) | -0.087(-0.165-0.010) | 0.210(0.156-0.288) | 0.003(-0.002-0.009) |
| Model 1 + Glycated albumin | 8747.793 | 8850.299 | 0.734(0.718,0.751) | 0.026(-0.004-0.072) | 0.214(0.094-0.324) | 0.000(-0.084-0.067) | 0.214(0.140-0.292) | 0.006(-0.001-0.014) |
| Model 2 | 10125.14 | 10224.76 |  | - | - | - | - | - |
| **Model 2 + hs-cTnT** | **9249.943** | **9353.346** | 0.746(0.729,0.763) | 0.106(0.059-0.190) | **0.564(0.400-0.669)** | **0.175(0.063-0.225)** | **0.390(0.298-0.474)** | **0.043(0.024-0.058)** |
| Model 2 + hs-cTnI | 9302.74 | 9406.189 | 0.765(0.748,0.782) | 0.046(-0.005-0.096) | 0.295(0.148-0.437) | 0.039(-0.061-0.112) | 0.256(0.162-0.383) | 0.019(0.008-0.031) |
| Model 2 + NT-proBNP | 9307.591 | 9411.009 | 0.757(0.74,0.775) | 0.086(0.031-0.145) | 0.381(0.236-0.513) | 0.158(0.072-0.242) | 0.222(0.141-0.313) | 0.032(0.014-0.050) |
| **Model 2 + Creatinine** | **10108.76** | **10213.63** | 0.76(0.743,0.777) | 0.032(-0.012-0.070) | **0.188(0.028-0.320)** | **-0.005(-0.086-0.065)** | **0.192(0.089-0.285)** | **0.010(-0.001-0.021)** |
| **Model 2 + Cystatin C** | **9347.044** | **9450.569** | 0.75(0.732,0.767) | 0.073(0.013-0.149) | **0.357(0.209-0.529)** | **0.022(-0.060-0.132)** | **0.335(0.197-0.436)** | **0.026(0.006-0.038)** |
| **Model 2 + β-2 microglobulin** | **9307.106** | **9410.585** | 0.76(0.743,0.777) | 0.107(0.023-0.143) | **0.413(0.261-0.559)** | **0.053(-0.034-0.149)** | **0.360(0.271-0.485)** | **0.031(0.007-0.046)** |
| Model 2 + Plasma glucose | 4574.251 | 4665.387 | 0.762(0.745,0.78) | 0.009(-0.034-0.084) | 0.107(-0.057-0.330) | -0.080(-0.163-0.079) | 0.188(0.054-0.300) | 0.002(-0.006-0.017) |
| Model 2 + HbA1c | 10113.25 | 10218.12 | 0.758 (0.740-0.775) | 0.014 (-0.014-0.062) | 0.028 (-0.008-0.085) | 0.021 (-0.022-0.063) | 0.007 (-0.006-0.027) | 0.004 (-0.003-0.012) |
| Model 2 + Glycated albumin | 8697.23 | 8799.687 | 0.760 (0.742-0.777) | 0.231 (0.088-0.346) | 0.207 (-0.003-0.335) | 0.000 (-0.140-0.071) | 0.207 (0.118-0.303) | 0.007 (-0.001-0.016) |
| **RECODe** | 10696.39 | 10786.36 | 0.759(0.743,0.775) | - | - | - | - | - |
| **RECODe + hs-cTnT** | 9694.443 | 9788.264 | 0.776(0.759,0.792) | 0.081(0.033-0.141) | 0.458(0.306-0.582) | 0.094(-0.016-0.184) | 0.365(0.253-0.426) | 0.035(0.018-0.052) |
| **RECODe + hs-cTnI** | 9728.47 | 9822.345 | 0.769(0.753,0.786) | 0.010(-0.015-0.090) | 0.372(0.187-0.499) | 0.046(-0.061-0.137) | 0.326(0.200-0.405) | 0.019(0.008-0.031) |
| **RECODe + NT-proBNP** | 9751.989 | 9845.863 | 0.774(0.758,0.79) | 0.073(0.018-0.132) | 0.245(0.140-0.408) | 0.078(0.024-0.201) | 0.167(0.076-0.255) | 0.033(0.018-0.057) |
| **RECODe + Cystatin C** | 9743.469 | 9837.409 | 0.776(0.76,0.792) | 0.069(0.005-0.122) | 0.355(0.238-0.505) | 0.107(-0.002-0.199) | 0.248(0.192-0.323) | 0.036(0.020-0.056) |
| **RECODe + β-2 microglobulin** | 9703.428 | 9797.355 | 0.781(0.765,0.797) | 0.100(0.044-0.176) | 0.392(0.270-0.543) | 0.071(0.020-0.185) | 0.321(0.224-0.410) | 0.049(0.033-0.072) |
| **RECODe + Plasma glucose** | 4795.238 | 4878.036 | 0.758(0.737,0.78) | 0.009(-0.076-0.042) | 0.031(-0.153-0.243) | 0.008(-0.112-0.162) | 0.022(-0.081-0.156) | -0.001(-0.006-0.015) |
| **RECODe + Glycated albumin** | 9774.543 | 9868.443 | 0.764(0.748,0.781) | 0.007(-0.021-0.052) | 0.180(0.012-0.314) | 0.091(-0.002-0.171) | 0.089(-0.005-0.170) | 0.003(-0.001-0.012) |
| **Model 2 + hs-cTnT NT-proBNP + Creatinine + β-2 microglobulin+ Glycated albumin** | 9112.772 | 9236.707 | 0.776 (0.760,0.793) | 0.180 (0.113-0.245) | 0.577 (0.402-0.690) | 0.211 (0.086-0.269) | 0.367 (0.272-0.480) | 0.066 (0.045-0.089) |

Hs-cTnT: high-sensitivity Troponin T; hs-cTnI: high-sensitivity Troponin I; NT-proBNP: N-terminal pro-B-type natriuretic peptid.

Model 1:gender, age, race, BMI.

Model 2 :gender, age, race, BMI, education, activity, PIR, cotinine, drinking, hypertension, hyperlipidemia, CVD, CKD.

**RECODe:** gender, age, race, clinical features (tobacco smoking, systolic blood pressure, cardiovascular disease history), drug use, biomarkers (HbA1c, total cholesterol, HDL cholesterol, serum creatinine, urine albumin: creatinine ratio).

**Supplement Figure 1**


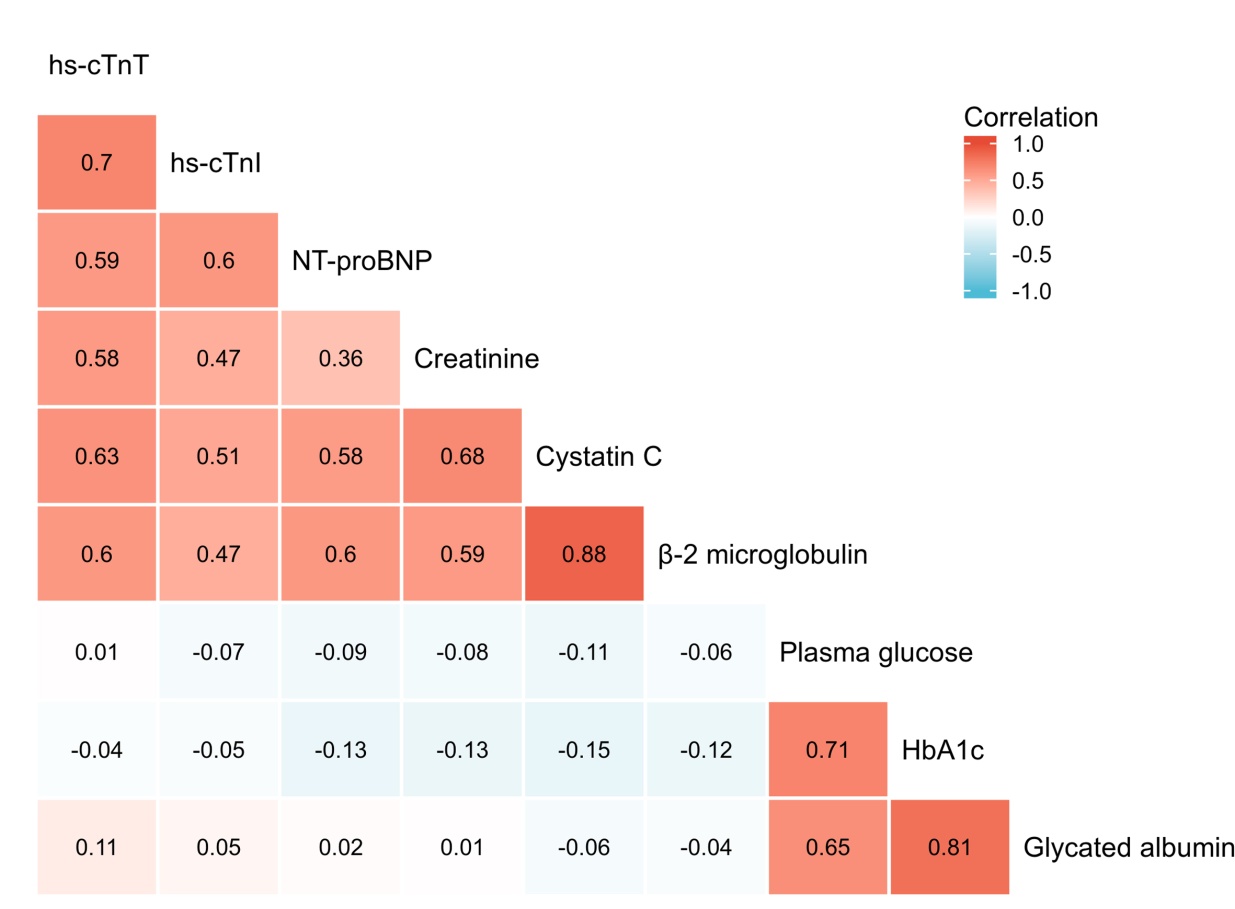

Supplement: Supplementary file 1 — Supplementary Material 1: Supplement Table 1. All-cause and Cause-Specific Mortality rates per 1000 person years [95% Confidence Interval (CI)]. Supplement Table 2. Comparison of Model Performance in Predicting All-Cause Mortality. Supplement Figure 1. A heat map shows the correlations among different biomarkers including hs-cTnT, hs-cTnI, NT-proBNP, creatinine, cystatin C, and β-2 microglobulin in a population of diabetic patients. The strength of the correlations ranged from near 0 to 0.81, indicating complex interrelationships that may arise from shared physiological pathways, underlying disease mechanisms related to diabetes, or common confounding factors such as age, gender, and co-morbidities. These findings highlight the need for a comprehensive understanding of the relationships among biomarkers in diabetic individuals for effective management and care. [file 12933_2023_1986_MOESM1_ESM.docx]
